# Kallikreins as Biomarkers for Prostate Cancer

**DOI:** 10.1155/2014/526341

**Published:** 2014-04-07

**Authors:** Sung Kyu Hong

**Affiliations:** Department of Urology, Seoul National University Bundang Hospital, 82 Gumi-Ro, 173 Beon-Gil, Bundang-Gu, Seongnam-Si, Gyeonggi-Do, Seongnam 463-707, Republic of Korea

## Abstract

The introduction of testing for prostate-specific antigen (PSA), a member of the fifteen-gene family of kallikrein-related peptidases and also known as kallikrein-related peptidase 3 (*KLK3*), in blood has revolutionized both the detection and management of prostate cancer. Given the similarities between PSA and other *KLK* gene family members along with limitations of PSA as a biomarker for prostate cancer mainly in reference to diagnostic specificity, the potential roles of other members of this gene family as well as PSA derivatives and isoforms in the management of prostate cancer have been studied extensively. Of these, approaches to measure distinct molecular forms of PSA (free, intact, complexed PSA, and pro-PSA) combined with kallikrein-related peptidase 2 (*KLK2*), also known as hK2, have been considered holding particular promise in enhancing the diagnosis of prostate cancer. Recently, an integrated approach of applying a panel of four kallikrein markers has been demonstrated to enhance accuracy in predicting the risk of prostate cancer at biopsy. This review presents an overview of kallikreins, starting with the past and current status of PSA, summarizing published data on the evaluations of various *KLKs* as biomarkers in the diagnosis, prognostication, and monitoring of prostate cancer.

## 1. Introduction

Tissue kallikrein or kallikrein-related peptidase 1 (*KLK1*) is a member of the chymotrypsin family of serine proteases. Since the first identified member of the* KLK* family was the most common protease in pancreas, the name of “kallikrein” is derived from pancreas (kallikreas) in Greek [1, 2]. Subsequently, the* KLK* family of serine protease was defined with the identifications of novel* KLKs, KLK2* (also known as human kallikrein 2: hK2), and* KLK3* (also known as prostate-specific antigen: PSA) [1–3]. Several investigators contributed to the identification of 12 additional novel serine protease genes localized in close proximity to the previously identified* KLK*-encoding genes that overall encompass *≈*280 kb at chromosomal region 19q13.4 of the human genome [2].* KLK* genes share various features, including exon/intron organization, number and length of exonic regions, intron phase, positioning of the methionine start codon, the catalytic-triad residues, and the terminal codons [4]. As the expression of* KLKs* has been detected in tissues and cell lines from many different human organs,* KLK* gene family members have been implicated in a broad spectrum of physiological processes, including blood pressure regulation, skin desquamation, seminal clot liquefaction, tissue remodeling, peptide hormone, and processing and inflammatory cascades [4].

The interest in* KLKs* as biomarker for prostate cancer dates back more than three decades ago when investigators first reported on the ability to detect PSA in serum from prostate cancer cases [5]. Due to the structural similarities between PSA and other* KLKs*, possible roles for the other members of* KLK* family as a biomarker for prostate cancer have also been explored during the past 25 years. This review strives to provide an overview on the clinical applications of PSA and other* KLKs* as the diagnostic and prognostic markers in prostate cancer.

## 2. History of PSA

In the 1930s, prostatic acid phosphatase (PAP) was first observed to be elevated in the serum of men with metastatic prostate cancer [6]. In the seminal report by Huggins and Hodges, PAP activity was used to indicate the success or failure of hormonal therapy, and, for close to 50 years, PAP was used as a blood biomarker of disease progression for men with advanced stages of prostate cancer [7]. However, attempts to use PAP for early detection of prostate cancer were not successful [8]. Accordingly, the diagnosis of prostate cancer remained a purely clinical endeavor for a long time.

Then, initially identified in 1966, PSA (*KLK3*), a 33-kDa glycoprotein secreted by prostatic epithelial cells, was first characterized in 1971 by Hara et al. in forensic studies as a marker for human semen [9]. Synthesized in prostate tissue, the proteolytic activity of the active PSA-enzyme catalyzes the degradation of the gel-forming proteins (*SEMG1* and* SEMG2*) in the ejaculate contributed by the seminal vesicles, which results in liquefaction and release of motile sperm [10–13]. Normally confined within the prostate, only a minute fraction (*≈*10^−6^) of the concentration of PSA being present in semen (which ranges from about 10 to 50 *μ*mol/L) can normally be detected in the blood circulation (i.e., 0.5 to 1 ng/mL, which corresponds to *≈*15–30 pmol/L). Prostate cancer is histologically characterized by loss of basal cell layer, derangement of the basal lamina, diminished epithelial polarity, and lack of connection of the glandular acini [14]. Although the increased serum level of PSA in prostate cancer patients is commonly considered to be due to disruption of prostate architecture associated with, for example, cancer, the exact molecular mechanism by which serum level of PSA is increased in prostate cancer patients is still unclear as experimental models and data are currently lacking [15, 16]. The use of PSA as a biomarker for prostate cancer was first postulated in the 1970s, when it was isolated from normal, benign hypertrophic, and cancerous prostatic tissues by Wang et al. [17]. Subsequently, other studies recognized the potential value of PSA as a biomarker of prostate cancer, and PAP was rendered largely obsolete [18]. The detection of PSA in serum was first reported by Papsidero et al. [5]. By the mid-1980s, data were accumulated to show that PSA was superior to PAP in monitoring of prostate cancer after treatment, resulting in the approval of PSA serum concentration assay for the follow-up and monitoring of prostate cancer patients from the US Food and Drug Administration (FDA) in 1986 [19]. Several studies showed that PSA was a sensitive marker for detecting residual disease and recurrence after treatment of prostate cancer during follow-up as we still consider an undetectable level of PSA after radical prostatectomy as indicating the absence of postoperative recurrence [20–22]. Similarly, serial PSA measurements were also applied in the contemporary setting to define recurrence following definitive radiation treatment [18, 23].

Subsequent studies in the early 1990s suggested that serum PSA may be useful in early detection of prostate cancer [18, 24, 25]. In a clinical trial of 6,630 men, Catalona et al. showed that combination of PSA ≥ 4.0 ng/mL with other clinical findings, such as those from digital rectal examination (DRE), improved the detection of prostate cancer detection [26]. In consideration of these findings, FDA also approved the usage of PSA for early detection of prostate cancer. This enabled PSA screening to become widely adopted and also led to a dramatic increase in prostate cancer incidences during mid-1990s in the USA [27]. Furthermore, the percentage of patients with clinically localized, early-stage disease increased substantially as documented in the Surveillance, Epidemiology, and End Results (SEER) database [18, 28]. Such stage migration resulted in the decrease of prostate cancer-specific mortality in the USA as well [29].

As for the appropriate PSA threshold for a prostate biopsy, this issue has been debated intensely since the early introduction of PSA testing in the USA. Data reported from the randomly selected, untreated controls in the Prostate Cancer Prevention Trial (PCPT) who underwent end-of-study prostate biopsy showed that PSA levels in blood were associated with evidence of prostate cancer at biopsy (AUC: 0.68) but that there was no PSA threshold in men aged 62–91 below which the presence of any prostate cancer lesion can be ruled out [30]. PSA's ability to detect the presence of Gleason grade 4 cancers at biopsy was higher (AUC: 0.75), but similarly there was no PSA level below which the presence of high grade cancer at biopsy could be excluded with reliable accuracy. With regard to applying the traditional cutoff of 4.0 ng/mL for triggering a biopsy, it has previously been reported that a significant proportion of tumors detected may be already spread to prostatic capsule at the time of diagnosis [31]. Also, it has been reported that 22% of men with a normal DRE and a serum total PSA level between 2.6 and 4.0 ng/mL have prostate cancer [32]. In addition, findings from PCPT revealed that about 15% of men with normal DRE and a serum total PSA less than 4.0 ng/mL may harbor prostate cancer [30]. As such, some advocated decreasing the threshold for biopsy to 2.5 ng/mL [18, 32, 33]. Meanwhile, aforementioned report from PCPT actually showed that 17% of men with low PSA (1.1–2.0 ng/mL) and a normal DRE had prostate cancer, indicating that even the most stringent biopsy criteria would miss a significant proportion of cancers [30]. Another problem with lowering the PSA threshold for biopsy would be that PSA may be elevated as a result of various noncancerous conditions. About 75% of men undergoing biopsy due to PSA in the range of 4.0 to 10.0 ng/mL do not have evidence of prostate cancer at systemic sextant prostate biopsy [34]. Thus, a significant number of patients are rendered to unnecessary cost as well as stress and morbidity. Although lowering PSA threshold and/or decreasing the age for PSA screening may well be beneficial for men who are at increased risk for prostate cancer, such as those with family history of prostate cancer, the determination of the optimal management-guiding threshold for general population should involve not only the clinical and epidemiologic features but also the social and psychological implications [33].

## 3. Controversy in PSA Screening

Despite the view that PSA screening contributed to significant decrease in prostate cancer-specific mortality rate in 1990s, recent studies have shown that PSA screening may lead to the unnecessary detection and treatment of indolent cancers, which may be associated with significant morbidity for the patients [35, 36]. Hence, the question on the usefulness of PSA screening remains. PSA screening came under increased scrutiny when two largest prospective screening trials to date demonstrated contradictory findings. Preliminary results of the Prostate, Lung, Colorectal, and Ovarian Cancer Screening Trial (PLCO) in the United States comparing annual screening to usual care found no significant difference in cause-specific mortality, although there were numerous methodological limitations to this study [37]. Some of the problems mentioned regarding the PLCO trial include relatively smaller sample size, the higher rate of PSA testing in the control arm (52%), the higher rate of prior history of PSA testing, and lower compliance with prostate biopsy among men with an elevated PSA level [14, 37–39]. In the European Randomized Study of Screening for Prostate Cancer (ERSPC) trial, 182,000 men aged 50–74 years were enrolled from 7 European countries [39]. For this trial, measurement of PSA level, with a threshold for prostate biopsy of 3 ng/mL, was the principal screening method. The interim data from 162,000 men during a median follow-up period of 9 years demonstrated that PSA screening reduced the risk of death due to prostate cancer by 20%. However, ERSPC trial also revealed that screening was associated with substantial overdiagnosis as it was observed from the trial that 1,410 men would need to be screened and 48 additional cases of prostate cancer would need to be treated to prevent one death from the disease. When the noncompliance among the men who were actually screened was adjusted for, screening was assessed to have reduced mortality from prostate cancer by 27% [39]. Overall the performance of PSA testing as a screening tool for prostate cancer is known to be variable. Depending on the PSA cutoff values applied, the specificity and sensitivity of PSA range from 20 to 40% and 70 to 90%, respectively [40]. The area under the curve (AUC) of the receiver operating characteristic (ROC) curve is between 0.55 and 0.70 for the ability of PSA to identify prostate cancer [40, 41]. As aforementioned, one of the explanations for such poor specificity is the fact that several noncancerous causes may increase PSA level. Due to high false-positive rate, PSA screening for prostate cancer demonstrates a positive predictive value of only 25 to 40% [42]. In 2002, the U.S. Preventive Services Task Force (USPSTF) issued a statement mentioning that the evidences are insufficient to recommend routine use of PSA as a screening tool among men younger than age 75 [40]. In 2011, the USPSTF reanalyzed the available evidence and concluded that the population benefiting from PSA screening was inconclusive, recommending against PSA screening at any age [43]. Thus the controversy continues on whether the benefits of PSA screening outweigh its risks.

## 4. Risk Stratification with PSA

Interestingly, several studies have demonstrated that PSA levels can be used to predict the future risk of prostate cancer, even decades before actual diagnosis [44–47]. It was reported from a large, prospective study that the leadtime between total PSA levels ≥ 4 ng/mL and the subsequent clinical diagnosis of prostate cancer was approximately 5.5 years [44]. Extending prediction models to lower PSA ranges and longer follow-up intervals, Lilja et al. reported from examining prostate cancer risk among 21,277 men younger than 50 years that PSA level checked at age 44 to 50 was very strongly associated with the likelihood of developing prostate cancer up to 25 years later [45]. Odds ratio for a prostate cancer diagnosis at a PSA level in the range of 0.51–1.0 ng/mL was 2.51 compared to PSA ≤ 0.50 ng/mL. Also odds ratio increased to 7.02 for a PSA of 1.0–1.5 ng/mL and to 19.01 for 2.01–3.0 ng/mL compared with PSA ≤ 0.50 ng/mL. In a subsequent study, the same group demonstrated that PSA level at age 44–50 predicted the risk of developing advanced prostate cancer [46]. Another study from the same group found that PSA at age of 60 years is an extremely strong predictor of the risk of prostate cancer metastasis (AUC 0.86) and death (AUC 0.90) by age 85 [47]. In this study, 90% of total mortality occurred in men with PSA > 2 ng/mL, whereas men with PSA < 1 ng/mL had 0.5% risk of metastasis and 0.2% risk of death from prostate cancer by the age of 85. Such finding would indicate that at least half of men can be exempted from PSA screening at age 60, which would allow the early detection efforts to focus on men with elevated risk [33]. Overall, these data show that men who will develop prostate cancer in future have elevated PSA levels many years before the diagnosis.

## 5. Strategies to Enhance the Accuracy of PSA

As aforementioned, PSA is not a perfect biomarker for the diagnosis of prostate cancer. Hence, the efforts have been made to enhance the diagnostic accuracy of PSA through PSA kinetics. Increasing the specificity would reduce the number of prostate biopsies performed and related burden in men without prostate cancer. One of the tools devised for such purpose is PSA velocity (PSAV). PSAV is defined as the serial evaluation of serum PSA levels over time [48, 49]. PSAV can be calculated via different methods, such as using the first and the last measured values only or applying a regression line through all available measurements. Carter et al. reported that PSAV is only useful if a minimum of 3 consecutive PSA measurements were taken over a two-year period [50]. Prospective studies have found that PSAV does not appear to add diagnostic value for prostate cancer detection beyond that of a single PSA measurement. In a landmark trial of PCPT, it was observed that when PSAV was adjusted for the effect of PSA and other standard variables, it lost its independent predictive value as an independent predictor of prostate cancer [51]. Meanwhile, the PLCO trial showed that although PSAV was an independent predictor of high grade prostate cancer, addition of PSAV only slightly enhanced the prediction of high grade tumor [52]. From analyzing a large cohort of men in early middle age who were likely to have a low incidence of BPH, Ulmert et al. calculated the predictive values of PSAV alone and a single PSA alone for diagnosis of prostate cancer to be 71.2% and 77.1%, respectively, demonstrating no benefit for PSAV [53]. Such result also indicates that PSA levels generally do not rise sharply before the detection of prostate cancer.

Some researchers have shown that a high pretreatment PSAV is strongly associated with lethal disease demonstrating poor survival following diagnosis. In the Baltimore Longitudinal Study of Aging project, a strong association between survival and higher PSAV as early as 10 to 15 years before diagnosis was observed [54]. Based on such findings, a total PSAV threshold of 0.35 ng/mL/year was also proposed to be used in screening men with low PSA levels to increase the detection of potentially lethal tumors still in the window of curability. However, Vickers et al. reported that performing biopsies in men with low PSA but elevated PSAV led to a large increase in unnecessary biopsies while missing a significant portion of clinically significant disease [55]. Also, it has been reported that, although relevant to prognosis, baseline total PSA levels and relative PSAV in the first two years following diagnosis of localized prostate cancer could not accurately predict which patients would have a lethal cancer-specific outcome [56]. In addition, several other studies have found that PSAV does not provide additive data in the prediction of prognosis following treatments [57, 58]. Potential reasons for such lack of accuracy may be that the observation period necessary for obtaining a valid calculation of PSAV that is not disturbed by considerable short-term fluctuations is too long or that the number of PSA measurements is too high for use in clinical practice [33]. And it just may be that PSAV does not correlate with early tumor progression but could be a mere indicator of aggressive disease which would not be considered curable even with early detection. Furthermore, a rapid elevation of PSA level is certainly a phenomenon more common in men with a high starting PSA level [59]. However, such situation will not be common scenario for men in a screened cohort. Despite the reported findings indicating the usefulness of PSAV and the suggestions that PSAV should be a part of guidelines and inclusion criteria for clinical trials, more evidences are necessary to confirm clinical utility of PSAV [14]. It should be taken into account that most of relevant studies, in reality, did not include PSAV with PSA in the multivariate predictive models [60].

Another method developed to enhance the predictive value of PSA is PSA doubling-time (PSADT), which is defined as the time it takes for serum PSA level to double. Initial studies on PSADT showed its potential utility in discriminating the biological significance of tumor recurrence after treatment [18]. Pound et al. reported that a postoperative PSADT <10 months was associated with worse metastasis-free survival [61]. Subsequent studies demonstrated that lower PSADT was associated with increased risk of cancer-specific mortality in men with PSA recurrence after surgery and radiation therapy [62, 63]. Still, controversy continues regarding the utility of PSADT as well as PSAV. Although some researchers have mentioned that PSA kinetics in the forms of PSAV and PSADT has improved the prognostic value of PSA measurements, a systematic review by Vickers et al. indicated that PSAV or PSADT provided little additive information over that offered by PSA level alone [18, 60]. Also the EAU guidelines state that PSAV and PSADT have limited use in the diagnosis of prostate cancer due to BPH, variations in interval between PSA measurements, and acceleration/deceleration of PSAV and PSADT over time [64].

Another modality devised to enhance the diagnostic accuracy of PSA was PSA density (PSAD), the concept of which was first described by Benson et al. as the ratio of serum PSA level to prostate volume [65]. This initial study identified the potential utility of PSAD in differentiating prostate cancer from benign disease with subsequent studies demonstrating a modest improvement in diagnostic accuracy with application of PSAD in addition to PSA [66]. Since the transition zone is primarily involved in BPH, several investigators studied the value of adjusting PSA to transition zone volume rather than total prostate volume [67]. Despite some positive findings reported, PSAD failed to receive wide acceptance in screening setting [68]. Other studies have demonstrated the utility of PSAD in predicting clinicopathological features of disease, such as Gleason score and total cancer volume at radical prostatectomy [69]. Overall as some reported that PSAD may contribute to predicting the risk of progression in men on active surveillance, PSAD may be most effective when used in conjunction with other clinical factors to stratify risk and weigh treatment options [70]. A significant problem with PSAD is the need for transrectal ultrasound, which is not a routine part of screening process and often not done as a separate procedure prior to biopsy. Also transrectal ultrasound measurement of prostate volume is prone to be affected by interexaminer variability. Meanwhile, a recent study demonstrated that a new ERSPC risk calculator incorporating prostate volume based upon DRE provided comparable predictive accuracy in predicting significant prostate cancer with transrectal ultrasound-based risk calculator (AUC 0.85 versus 0.86) [71]. Such replacement of transrectal ultrasound measurements with DRE estimates may well enhance the implementation of PSAD as well as prostate volume into risk stratification in clinical setting.

## 6. PSA Subforms and Human Kallikrein 2 (hK2)

In 1991, Lilja et al. reported that the major immune-detected fraction of PSA in serum exists in complex with protease inhibitor *α*1-antichymotrypsin [72]. Also, Lilja et al. identified a distinct PSA-epitope present only on the free, noncomplexed, minor fraction of total PSA in circulation. PSA that is catalytically inactive does not form complexes and circulates as free PSA (fPSA). Distinct isoforms of fPSA will be discussed separately in the following section. Levels of fPSA can be detected and compared to total PSA level, yielding the proportion of fPSA (%fPSA). Studies have shown that men with the highest proportions of complexed PSA are more likely to have prostate cancer and that %fPSA is lower in men with prostate cancer as compared to BPH [18, 73, 74]. In 1995, Luderer et al. reported from performing a comparative study that %fPSA outperformed PSA in the “diagnostic gray zone” (PSA: 4.0–10.0 ng/mL) [75]. In a study of men with intermediate PSA levels, a fPSA threshold of 25% yielded 95% sensitivity and 20% specificity for prostate cancer diagnosis [76]. Meanwhile, the fPSA threshold of 20% improved specificity to 38% among men with PSA in the range of 3.0–7.0 ng/mL. In 1998, a prospective study demonstrated that %fPSA decreased the rate of unnecessary biopsies by 20% when using a threshold fPSA of 25% and that, in general, cancers detected at fPSA > 25% were of smaller volume and lower grade [77]. Such findings were also observed in other studies most commonly implementing %fPSA thresholds in the range of 20 to 27% [78]. In consideration of these published findings, %fPSA was approved by the FDA as an adjunct to PSA for use in the screening and diagnosis of prostate cancer in men with PSA levels between 4.0 and 10.0 ng/mL. Some studies indicated that measuring %fPSA may increase diagnostic accuracy in cases presenting with either low or high PSA values. A randomized, population-based study demonstrated that low %fPSA in men with PSA levels < 3 ng/mL was associated with a 5- to10-fold increased risk of prostate cancer [79]. Also the %fPSA was observed to increase the accuracy of prediction of biopsy results in men with PSA in the range of 10–20 ng/mL [80]. Subsequent studies identified potential shortcomings in using fPSA, such as higher %fPSA in larger prostates, conditions at the time of sample obtainment, in vitro instability, and interassay variability, which may help explain the inconsistencies in the performance of fPSA [18]. Also, published data on the usefulness of fPSA in predicting clinical and pathological outcomes following surgical treatment have shown inconclusive results [81, 82]. However, a meta-analysis of 66 published studies concluded that the %fPSA outperforms PSA in the detection of prostate cancer [83]. Despite some limitations, overall fPSA appears to be a useful modality for diagnosis of prostate cancer, particularly in men with intermediate PSA levels [18].

Human kallikrein 2 (hK2: also known as human kallikrein-related peptidase 2), a secreted serine protease sharing an 80% sequence homology with PSA, is responsible for the cleavage of pPSA to active mature PSA [84, 85]. Both hK2 and PSA are primarily expressed in the prostate gland [16]. Despite these similarities, hK2 and PSA differ in their enzymatic activity [33]. Since the covariance of hK2 and PSA is less than 60% and both markers demonstrate different expression patterns on an immunohistochemistry level, hK2 is considered a marker independent of PSA [86, 87]. The levels of hK2 in prostate, semen, and serum are less than 2% compared with PSA. Similar to PSA, serum hK2 is present in two forms in the blood: one bound to various protease inhibitors and the other (preponderant) free in the circulation [33]. Initial reports showed that although prostate cancer and BPH patients showed no significant difference in hK2 levels, the ratio of hK2 to fPSA (%hK2) increased the accuracy over %fPSA in differentiating prostate cancer from BPH in men with PSA in the range of 4–10 ng/mL [88]. Subsequently, several groups reported that %hK2 contributed to an enhanced discrimination between prostate cancer and noncancer patients [89, 90]. However, others reported that predictive value of %hK2 may not be higher than %fPSA [91, 92]. Meanwhile, it has been suggested that hK2 could also be useful in predicting pathologic stage and grade along with biochemical outcome in patients treated with radical prostatectomy [93, 94]. However, this finding is yet to be validated as some observed that hK2 was unable to discriminate pathologic stage or failed to demonstrate additional value over existing variables [95, 96]. Overall most relevant studies concluded that hK2 has additive role in the detection of prostate cancer. More investigation is warranted on the potential role of hK2 as a prognostic marker.

There are three distinct cleavage isoforms of free PSA in the serum: BPH-associated PSA (BPSA), intact free PSA, and pro-PSA which has been associated with prostate cancer [97]. The precursor of PSA is a 261-amino acid preproprotein, and subsequent processing by human glandular kallikrein2 (hK2) produces active 237-amino acid mature PSA [33]. In men with PSA levels between 6.0 and 24.0 ng/mL, the [−2]pro-PSA fraction was found to be significantly higher in men with prostate cancer [98]. Furthermore, the same group later reported on the value of the pro-PSA to fPSA ratio for screening patients with PSA levels between 2.5 and 4.0 ng/mL and between 4.0 and 10.0 ng/mL [99]. For proper measurement of [−2]pro-PSA, blood samples should be centrifuged within 3 hours of sample collection [30]. Serum may be stored at room temperature or refrigerated (4°C) for a maximum of 48 hours and should be frozen, if stored for a longer period [33]. Two freeze-thaw cycles have no effect on [−2]pro-PSA stability [100]. Catalona et al. reported that elevated pro-PSA to fPSA ratios were associated with aggressive pathological features and decreased biochemical disease free survival after radical prostatectomy [101]. Stephan et al. demonstrated that a new automated tool using [−2]pro-PSA assay with a %fPSA based artificial neural network was capable of detecting prostate cancer and more aggressive disease with higher accuracy than PSA or %fPSA alone [102].

## 7. Combination of Kallikrein Markers for Improved Cancer Detection

Since it is unlikely that a single biomarker will be enough to make a clear-cut decision regarding the diagnosis and/or prognosis of prostate cancer, efforts have been made to also evaluate the combination of a panel of complimentary biomarkers. A group led by Lilja and Vickers devised a statistical model for predicting prostate biopsy outcomes based on age, DRE, and a panel of four kallikrein markers which were total PSA, fPSA, intact PSA, and hK2. Analyzing the data from the randomized prostate cancer screening trial in Göteborg, Sweden, which was also a part of ERSPC trial, they reported that for every 1000 previously unscreened men with elevated PSA, the use of the model to determine whether to perform biopsy would reduce biopsy rates by 573, while missing only 31 of 152 low grade cancers and 3 of 40 high grade cancers [103]. Such results were also replicated via an independent cohort in which the use of model resulted in the reduction of biopsy by 513 per 1000 men with elevated PSA, whereas 54 of 177 low grade cancers and 12 of 100 high grade cancers were missed [104]. In men who recently have undergone previous screening, the use of the model demonstrated improvements in predictive accuracy [105, 106]. Recently, the panel of four kallikrein markers was shown to predict the outcome of prostate biopsy in men who had previously undergone prostate biopsy during previous screening [107]. In this study, utilizing the dataset obtained from ERSPC trial, the application of four-kallikrein panel significantly enhanced the predictive accuracy of a base model, incorporating age, DRE, and PSA. As the panel of four kallikrein markers is in the phase of being commercialized (OPKO 4Kscore Prostate Cancer Test), it may soon be available in clinical setting. Meanwhile, published studies have shown that the Beckman Coulter Prostate Health Index (phi), which is a mathematical combination of total PSA, fPSA, and p2PSA (aforementioned as [−2]pro-PSA), as well as p2PSA and its derivatives, namely, %p2PSA, defined as (p2PSA/fPSA) × 100, may significantly improve the accuracy in the detection of prostate cancer over the PSA and the %fPSA [15, 108, 109]. Recently, US FDA has approved the phi test as a new way to test for risk of prostate cancer in men with PSA values in the 4–10 ng/mL range. A head-to-head comparison of phi and PCA3 recently showed that phi was more accurate than PCA3 in both initial and repeat biopsy setting although no statistical significance was noted [110]. In this study, PCA3 did not increase the accuracy of predicting prostate cancer when phi was assessed.

## 8. Other Kallikreins

As they are also expressed in prostate tissue and are closely related to PSA and hK2, the other members of the tissue kallikrein family have also been investigated upon for their potential role in the detection and prognosis of prostate cancer. Some have reported that higher kallikrein-related peptidase 4 (*KLK4*) mRNA levels of the prostate tissue obtained via biopsy are correlated with higher Gleason score and stage [4]. Kallikrein-related peptidase 5 (*KLK5*) is overexpressed in normal versus cancerous prostatic tissue, and an inverse relationship has been reported between* KLK5* levels and pathologic tumor stage and grade [111, 112]. Also, some studies reported severe overexpression of* KLK5* gene transcription levels with treatment of the androgen-independent prostate cancer cell lines PC3 and DU145 with chemotherapeutic agents widely used in clinical setting [113, 114]. The observation that modulation of the expression levels of these genes was triggered by anticancer agents showed the potential value of* KLKs* in monitoring and evaluating therapeutic responses to chemotherapy in androgen-independent prostate cancer [4]. Meanwhile, elevated kallikrein-related peptidase 11 (*KLK11*) mRNA expressions have been found to be associated with a less advanced stage, lower Gleason score, and an optimistic disease course for prostate cancer [4]. Expression of kallikrein-related peptidase 14 (*KLK14*), which is likely to have a major role in seminal clot liquefaction, indicates an adverse clinical outcome of prostate cancer patients as elevated* KLK14* mRNA and protein levels have been associated with more aggressive tumors [4, 115]. The overexpression of kallikrein-related peptidase 15 (*KLK15*) transcript variants, including alternatively spliced ones, has been observed to be associated with more aggressive prostate cancer [116, 117]. Overall, despite the promising findings reported, further research would be needed to elucidate potential roles for these kallikreins as biomarkers for prostate cancer. Emerging and ongoing efforts on studying* KLK*-mediated pathways will provide further information to support evaluations of* KLKs* as potential biomarkers for prostate cancer. Although* KLKs* may individually lack sufficient specificity and/or sensitivity to be used as a useful biomarker, groups of* KLKs*, possibly with other markers, may offer enhanced accuracy.

## 9. Conclusions

Interests in kallikreins as biomarkers for cancer began with the advent of PSA which certainly opened up a new era in the management of prostate cancer. Decades following the introduction of PSA, controversy persists regarding the appropriate use of the biomarker. Continuous efforts have been made to improve the accuracy of PSA and/or develop new biomarkers for prostate cancer. Various forms of PSA dynamics failed to significantly enhance the predictive value of PSA. Meanwhile, an integrated approach of applying a panel of different molecular markers, namely, PSA and other* KLKs*, may hold the promise of improving the screening, diagnosis, and monitoring of prostate cancer. Despite the substantial advances in the understanding of* KLKs*, their role in the pathophysiology of prostate cancer is just beginning to be understood. With advances in genomics, proteomics, and other biotechnology, the actual roles of* KLKs* in prostate cancer will likely be elucidated in the near future to help provide novel biomarker for improving screening, diagnosis, prognostication, and eventually patients' survival.
